# Optimal H_2_O_2_ preconditioning to improve bone marrow mesenchymal stem cells’ engraftment in wound healing

**DOI:** 10.1186/s13287-020-01910-5

**Published:** 2020-10-08

**Authors:** Ling Guo, Juan Du, Dan-feng Yuan, Ya Zhang, Shu Zhang, Hua-cai Zhang, Jun-wei Mi, Ya-lei Ning, Min-jia Chen, Da-lin Wen, Jian-hui Sun, Di Liu, Ling Zeng, Anqiang Zhang, Jianxin Jiang, Hong Huang

**Affiliations:** State Key Laboratory of Trauma, Burns and Combined Injury, Department of Surgical Research, Daping Hospital, Army Medical University, Chongqing, 400042 China

**Keywords:** Mesenchymal stem cells, Hydrogen peroxide, Preconditioning, Oxidative stress, Wound healing

## Abstract

**Background:**

The transplantation of bone marrow mesenchymal stem cells (BMSCs) is a promising therapeutic strategy for wound healing. However, the poor migration capacity and low survival rate of transplanted BMSCs in wounds weaken their potential application.

**Objective:**

To identify the optimal protocol for BMSCs preconditioned with H_2_O_2_ and improve the therapeutic efficacy using H_2_O_2_-preconditioned BMSCs in wound healing.

**Methods:**

Mouse BMSCs were exposed to various concentrations of H_2_O_2_, and the key cellular functional properties were assessed to determine the optimal precondition with H_2_O_2_. The H_2_O_2_-preconditioned BMSCs were transplanted into mice with full-thickness excisional wounds to evaluate their healing capacity and tissue engraftment.

**Results:**

Treatment BMSCs with 50 μM H_2_O_2_ for 12 h could significantly enhance their proliferation, migration, and survival by maximizing the upregulation of cyclin D1, SDF-1, and its receptors CXCR4/7 expressions, and activating the PI3K/Akt/mTOR pathway, but inhibiting the expression of p16 and GSK-3β. Meanwhile, oxidative stress-induced BMSC apoptosis was also significantly attenuated by the same protocol pretreatment with a decreased ratio of Bax/Bcl-2 and cleaved caspase-9/3 expression. Moreover, after the identification of the optimal protocol of H_2_O_2_ precondition in vitro, the migration and tissue engraftment of transfused BMSCs with H_2_O_2_ preconditioning were dramatically increased into the wound site as compared to the un-preconditioned BMSCs. The increased microvessel density and the speedy closure of the wounds were observed after the transfusion of H_2_O_2_-preconditioned BMSCs.

**Conclusions:**

The findings suggested that 50 μM H_2_O_2_ pretreated for 12 h is the optimal precondition for the transplantation of BMSCs, which gives a considerable insight that this protocol may be served as a promising candidate for improving the therapeutic potential of BMSCs for wound healing.

## Introduction

Growing evidence indicates that BMSC-based therapy for cutaneous wound healing holds great therapeutic value via differentiating into specialized cell types, producing a large variety of growth factors, and promoting wound closure and angiogenesis [1–4]. Therefore, transplantation of BMSCs is a promising therapeutic strategy for wound healing [1]. However, the poor migration capacity and low survival rate of transplanted BMSCs in wounds weaken their potential application.

Preconditioning has become the most powerful and effective cytoprotective strategy for initiating cell survival signaling to counter the rigorous harsh microenvironment [5, 6] and enhance the cell migration capacity and survival rate [5, 7]. Reportedly, the injured tissue expresses a high level of stromal cell-derived factor (SDF-1), a stem cell homing signal factor which binds to its receptor CXC chemokine receptor 4 (CXCR4) and CXCR7 on cell surface of MSCs and promotes stem cells homing and survival in the injured tissue, respectively [7, 8]. However, during in vitro culture expansion, BMSCs lose their CXCR4 receptor [9] and reduce their binding capacity to SDF-1 resulting in [10, 11] attenuation of their migration capacity. Moreover, various stress conditions including ex vivo isolation, in vitro expansion of MSCs, and confronting the harsh microenvironment (ischemia, hypoxia, and inflammation) caused oxidative stress injury to BMSCs following engraftment at injured sites, resulting in reducing survival of transplanted BMSCs [12–14].

Reactive oxygen species (ROS)-mediated-oxidative stress is an important cause for apoptosis and death of donor MSCs following engraftment [5, 6, 12, 13]. Mechanistically, the phosphatidylinositide 3-kinases (PI3K)/protein kinase B (Akt) signaling pathway plays central regulatory roles in MSC survival, proliferation, migration, angiogenesis, cytokine production, and differentiation [15–17]. SDF-1 could promote MSC survival and migration by the activation of the PI3K/Akt pathway [9, 10, 18–21]. Several studies demonstrated that H_2_O_2_ preconditioning protects cells against oxidative damage [22–24]. In fact, H_2_O_2_ at certain low level acts as essential cellular messengers by playing a growth factor-like role in regulating physiological stem cellular processes [25–27]. Therefore, we speculated low-dose H_2_O_2_ functioned as a growth factor-like molecule to activate the PI3K/Akt pathway. Although multiple studies have confirmed the cytoprotective effect of H_2_O_2_ preconditioning on MSCs against oxidative damage, neither the optimal concentration of H_2_O_2_ used in preconditioning nor the underlying molecular mechanism has been fully established [22–24].

In this study, the optimal concentration of H_2_O_2_ on BMSC preconditioning was first determined. Then, the molecular mechanisms of H_2_O_2_ preconditioning BMSCs were explored by focusing on the PI3K/Akt pathway due to its role in MSC survival and migration responding to SDF-1 and oxidative stress [28]. The ultimate therapeutic potential of H_2_O_2−_preconditioned BMSCs was evaluated in a mouse full-thickness wound model.

## Materials and methods

### Ethics statement

Animal studies were performed using male Balb/C mice provided by the Animal Breeding Center of Third Military Medical University. All procedures in the present study were performed in accordance with the “Guide for the Care and Use of Laboratory Animals” (National Institutes of Health Publication no. 86-23, revised 1985) and were approved by our hospital’s ethics committee.

### MSC isolation, culture, and stimulation

Primary BMSCs were isolated from the long bones of 4-week-old male Balb/C mice as previously described [11]. For this purpose, the femurs and tibiae were aseptically removed from the euthanized animals and dissected clean of attached muscles. The bones were flushed with 2 mL of cold PBS and washed 1 time with PBS. Cells were suspended in DMEM/F12 (Hyclone, Logan, UT) and supplemented with 10% (vol/vol) fetal bovine serum (FBS, Invitrogen), 100 U/mL penicillin, and 100 μg/mL streptomycin, and cultured in 25-cm^2^ tissue culture flasks (Falcon) in a humidified atmosphere with 5% CO_2_ at 37 °C. After 24 h, non-adherent cells were depleted and 3 mL of fresh culture medium was added. After 7 to 10 days of initiation, BMSCs growing clone-like were passaged at 70 to 80% confluence using TrypLE™ Express (Gibco) digesting. Cells were washed and seeded at a density of 1 × 10^6^ in tissue culture flasks. BMSCs harvested between 3 and 7 passages were used for follow-up experiments.

### Cell ATP assay

Because mitochondrial activity can better reflect cell vitality, the cell viability was assessed using the ATP assay. A luciferase-based kit (Beyotime Biotechnology, Shanghai, China) was used to measure the ATP level according to the manufacturer’s instruction. Briefly, BMSCs were seeded into 96-well plates at a density of 1 × 10^4^ cells/well. After 24 h, cells were treated with various concentrations of H_2_O_2_ (0, 25, 50, 100, 200 μM, Sigma, St. Louis, MO, USA) for 24 h at 37 °C in a cell culture incubator. After that, the culture medium was removed and the cells in each well were lysed by lysis buffer and centrifuged (1200*g*, 4 °C, 5 min). Next, the supernatant was quickly mixed with the working solution at equal volumes and then transferred into a standard opaque well 96-well plate before light recording a microplate reader (Thermo, Varioskan LUX, USA) using the chemiluminescence method.

### Cell Counting Kit-8 assay

Cell Counting Kit-8 (CCK-8, Beyotime Biotechnology, Shanghai, China) was applied for the evaluation of cell proliferation. BMSCs were trypsinized and seeded in 96-well plates at a density of 1 × 10^4^ cells/well. After overnight incubation, the cell culture medium was replaced and simultaneously added with various concentrations of H_2_O_2_ (25, 50, 100, 150, and 200 μM). H_2_O_2_ was diluted with DMEM/F12 and used immediately. After that, 10 μL of CCK-8 solution was added into each well. Cells were incubated at 37 °C for 2 h in the dark. Absorbance values at the wavelength of 450 nm were detected using a microplate reader (Thermo, Varioskan LUX, USA). Each experiment was repeated 3 times.

### Scratch wound-healing migration assay

BMSCs were seeded in 6-well plates at a density of 1 × 10^5^ cells/well. When BMSCs grew to 90% confluence, scratches were made with a standard 200-μL plastic pipette tip to make a line-shaped wound across the diameter of each well. The medium was then aspirated, and the cells were washed thoroughly with PBS. The PBS was then aspirated. And BMSCs were incubated further for 12 h and 24 h with or without 50 μM H_2_O_2_. Images were captured at 0, 12, and 24 h using the phase-contrast microscope with × 20 magnification (Olympus, Japan). Five images were taken per scratch which represents a technical replicate of 5 and biological replicate of 3 for the stem cells. The cells within the scratch zone per field were counted using ImageJ software (National Institutes of Health, Bethesda, MD, USA).

### Cell migration

Migration assays were carried out in a 24-well transwell migration system with an 8-μm pore size polycarbonate membrane at a density of 2 × 10^4^ cells/well. BMSCs, preconditioned or not with 50 μM H_2_O_2_ for 12 h after incubating with or without CXCR4 antibody for 2 h, were plated in the top Boyden chamber (Corning Costar). The cells were allowed to migrate for 12 h in the presence of 50 ng/mL SDF-1 (Sigma, US) in the bottom chamber. The migrating cells underside of the membrane were fixed in 4% paraformaldehyde for 15 min, followed by staining with 0.5% crystal violet. The number of migrating cells was determined by counting five random fields per membrane under the microscope at × 400 magnification (Olympus, Japan) and represented as the average of three independent experiments.

### Flow cytometry analysis of BMSC apoptosis

Cell apoptosis was detected with Annexin V-fluorescein isothiocyanate (FITC)/propidium iodide (PI) Apoptosis Detection Kit (KeyGen Biotech, Nanjing, China) as described by the manufacturer’s instructions. Briefly, BMSCs were seeded in 6-well plates at a density of 1 × 10^5^ cells/well, and treated with various concentrations of H_2_O_2_ (25, 50, 100, 150, 200, 300, and 500 μM) for 24 h or treated with high concentrations of H_2_O_2_ (200, 250, 300, and 500 μM) for 24 h after preconditioned with or without 50 μM H_2_O_2_ for 12 h, or cells were exposed to 12-h preconditioning with H_2_O_2_ after a 2-h pre-incubation with or without PI3K inhibitor LY294002 (Sigma, US), followed by treatment with 300 μM H_2_O_2_ for 24 h (Table 1). Thereafter, cells were collected and washed with ice-cold PBS twice, gently resuspended in the binding buffer and incubated with annexin V-FITC and propidium iodide (PI) for 15 min in the dark. Cells were analyzed by flow cytometry (NovoCyte™, ACEA Biosciences, San Diego, USA). The total apoptosis rate of cells was calculated as the sum of the rates of cells observed in the lower-right quadrant and the upper-right quadrant.

**Table 1:** The BMSC-treated protocol for the flow cytometry analysis of apoptosis

Cell density (cells/well)	H_2_O_2_ (μM) procondition/time	LY294002 (μM) pre-incubation/time	H_2_O_2_ (μM) treatment/time
1 × 10^5^	No	No	25, 50, 100, 150, 200, 300, 500/24 h
1 × 10^5^	50 μM/12 h	No	200, 250, 300, 500/24 h
1 × 10^5^	50 μM/12 h	50 μM/2 h	300/24 h

### Osteogenic, chondrocytic and adipogenic differentiation

BMSCs were pretreated with 50 μM H_2_O_2_ for 12 h before the original medium was changed to a differentiation medium. StemPro Osteogenesis Differentiation Kit, StemPro Chondrogenesis Differentiation Kit, and StemPro Adipogenesis Differentiation Kit (Gibco) were used for osteogenesis, adipogenesis, and chondrogenesis, respectively. Differentiation was assessed using alkaline phosphatase, Toluidine blue, Alcian blue, and Oil Red O stain, respectively.

### Mitochondrial superoxide O_2_^• −^ assessment

The generation of mitochondrial O_2_^•−^ was determined using MitoSOX Red (Invitrogen, Carlsbad, CA, USA), a selective fluorescent indicator for detecting O_2_^• −^ generation within mitochondria. Briefly, BMSCs were seeded in 24-well tissue culture plates (2.0 × 10^4^ cells per well) and randomly divided into 4 groups: the control group, 50 μM H_2_O_2_ group, 300 μM H_2_O_2_ group, and 50 μM H_2_O_2_ plus 300 μM H_2_O_2_ group, in which cells were incubated for 24 h with medium only, 50 μM H_2_O_2_, 300 μM H_2_O_2_, and 300 μM H_2_O_2_ before preconditioned with 50 μM H_2_O_2_ for 12 h, respectively. Thereafter, the MitoSOX Red reagent stock solution was diluted with HBSS/Ca/Mg buffer to make a working solution of 5 μM. And cells were incubated with 5 μM MitoSOX Red reagent in the dark at 37 °C for 10 min. Cells were washed gently three times with warm buffer. Fluorescence intensities of mtROS were determined using a microplate reader (Thermo, Varioskan LUX, USA), and images were visualized by fluorescence microscopy (Olympus, Japan) with the emission wavelength of 579 nm and the excitation wavelength of 510 nm.

### Mitochondrial membrane potential (Δ*ψm*) determination

Mitochondrial membrane potential (Δ*ψm*) was detected using the fluorescent, lipophilic dye, JC-1 (Beyotime Biotechnology, Shanghai, China). JC-1 can easily penetrate cells with healthy mitochondria. A green fluorescent JC-1 probe exists as a monomer at low membrane potentials. However, at higher potentials, JC-1 forms red-fluorescent “J-aggregates.” BMSCs were trypsinized and seeded in 24-well tissue culture plates (2.0 × 10^4^ cells/well), randomly divided into 4 groups, and treated like as *2.9*. Thereafter, the cells were incubated with JC-1 staining solution for 20 min, then cells were washed twice with JC-1 staining buffer (1×). When the JC-1 monomer was detected, the excitation wavelength was set to 490 nm and the emission wavelengths was set to 530 nm. When JC-1 polymer was detected, the excitation wavelength was set to 525 nm and the emission wavelength was set to 590 nm using a microplate reader (Thermo, Varioskan LUX, USA). The Δ*ψm* was represented as the ratio of red to green fluorescence intensity. Then, these cells were observed and taken a photograph by fluorescence microscopy (Olympus, Japan).

### Wound healing model and BMSC transplantation

Animal studies were performed using 6-week-old male BALB/C mice (21–23 g) provided by the Animal Center of The Third Military Medical University. Animals were individually housed in plastic cages at 25 °C with access to food and water ad libitum. Animals were kept on a 12-h light/dark cycle. The full-thickness excisional skin wounds in Balb/C mice were made as previously reported [11]. Animals were weighed and anesthetized by intraperitoneal injection of the appropriate dose (60 mg/kg) of 2% pentobarbital sodium. After shaving the dorsal surfaces of mice, full-thickness excisional skin wounds (8 mm in diameter) were created aseptically on the midline of the mice’s back. Mice were randomly divided into three groups, which received an injection of a suspension of 100 μL PBS, 1 × 10^6^ BMSCs in 100 μL PBS and 1 × 10^6^ H_2_O_2_-preconditioned BMSCs (for 12 h) in 100 μL PBS, respectively. BMSCs were pre-labeled with chloromethyl-1,1-dioctadecyl-3,3,3,3-tetramethylindocarbocyanine perchlorate (CM-DiI, Sigma) before injection. BMSCs were incubated with 10 μM DiI for 20 min at 37 °C. Mice post-wounding at once were injected with DiI-labeled BMSCs or PBS via the tail vein.

### Wound analysis

Wound analysis began at 1 day after wounding. Digital photographs of wounds were taken on days 0, 1, 3, 5, 7, 10, 13, and 15 and analyzed using the ImageJ software. The wound closure rate was calculated as follows: [(area of original wound − area of actual wound)/area of original wound] × 100%.

Mice were sacrificed at days 0, 3, 5, 7, and 10 post-wounding, and the skin samples including the wound and a 4-mm marginal unwounded skin from each wound were collected for biochemical and histological analyses. Half of each sample was fixed in 4% paraformaldehyde for 5 h and then preserved in 20% sucrose at 4 °C or fixed in 4% paraformaldehyde for 12 h and embedded in paraffin for hematoxylin and eosin (H&E) staining, Masson’s trichrome stain, and CD31 immunohistochemical staining. These samples (preserved in 20% sucrose) were cut into 10-μm frozen sections and observed with a fluorescence microscope. The photos were taken by randomly selecting 6 high-power fields (hpf, × 200). We used the ImageJ software to calculate the total number of migrated BMSCs with CM-DiI label (red cells) at the wound site. The other half of the same sample was snap-frozen in liquid nitrogen and stored at − 80 °C until protein analysis using Western blot assay. Three animals were collected from each experimental time point.

### Western blot analysis

BMSCs seeded in 6-cm tissue culture dishes (1 × 10^6^/well) in the logarithmic growth phase were treated with various low concentrations of H_2_O_2_ (0, 25, 50, 100, and 200 μM) for 24 h or were treated with 50 μM for different time courses (0, 3, 6, 12, 24, and 36 h). For another set of experiments, either a 12-h pre-incubation with 50 μM H_2_O_2_ or with or without a 2-h pre-incubation with 50 μM PI3K inhibitor LY294002 (Sigma), followed by treatment with 300 μM H_2_O_2_ for 24 h, cultured cells were then collected. Total protein lysates of cultured cells and wound tissues were prepared using RIPA lysis buffer (Beyotime Biotechnology, Shanghai, China), and the protein concentrations were determined using a BCA Protein Assay Kit (Keygen, Nanjing, China). Equal volumes of proteins (50 μg) from each sample were separated on SDS-PAGE (4–20%) gels and transferred to PVDF membranes (Millipore, Billerica, MA). Then, the membranes were blocked with 5% non-fat dry milk in Tris-buffered saline for 60 min at room temperature and incubated with primary antibodies: cyclin D1, p16, SDF-1, CXCR4/7, Bcl-2, Bax, caspase3/9, cleaved-caspase3/9, PI3K/Phospho-PI3K, Akt/Phospho-Akt (pAktThr308), mTOR/Phospho-mTOR (Ser 2481), VEGF (Cell Signaling Technology, USA), β-actin (Sigma-Aldrich), and GAPDH (Abcam, Cambridge, UK) overnight at 4 °C, followed by sequential incubation with HRP-conjugated secondary antibodies for 2 h at room temperature. Lastly, the bands were visualized by an enhanced chemiluminescence detection kit (ECL, Amersham Arlington Heights, IL, USA) and exposed to the gel imaging system (Bio-Rad, USA). Band density was analyzed using LabWorks 4.6 analysis software.

### Microvessel density of the wound area

Microvessel density was determined by immunohistochemical staining with anti-CD31 antibody (Santa Cruz), according to the manufacturer’s instructions and a method previously described [11]. In brief, antigen retrieval was performed in a 0.01-mol/L citrate buffer (pH 6.0) with a heating interval—keeping the temperature at 92–98 °C by using a microwave oven for 15 min and then cooling for 30 min. The sections were permeabilized with 0.1% Triton X-100 (Sigma) for 10 min and incubated with 3% H_2_O_2_ in methanol for 20 min. After blocking with goat serum at room temperature for 1 h, the sections were incubated overnight at 4 °C with anti-mouse CD31 antibody diluted in 0.1% BSA in PBS. Subsequently, the sections were incubated with biotin-labeled secondary antibody for 30 min and then with streptavidin peroxidase for 30 min at 37 °C. The cell nucleus was counterstained with hematoxylin. The microvessels that were lined with the endothelium and had a patent lumen were counted. The mean number of microvessels, defined as CD31 positive, were counted using five high-power fields (5 hpf, × 400) per section using the ImageJ software. All measurements were performed under double-blind conditions.

### Analysis of collagen deposition and H&E staining in the wound area

To determine the extent of collagen deposition in the granulation tissue of the wounds at days 7 after injury, collagen was stained with Masson’s trichrome stain. We used the ImageJ software to quantify collagen deposition from the Masson’s trichrome stained images. Each image was captured under × 40 magnification by a blinded individual. Under the analyze function in ImageJ, we used the RedGreen Blue (RGB) histogram to determine the mean value for the blue color intensity, in which our trichrome images represent collagen. In addition, these sections of the granulation tissues of the wounds in each group at days 5 post-wounding were analyzed using H&E staining.

### Statistical analysis

We obtained all the results from at least three independent experiments. All statistical analyses were performed using the SPSS13.0 software. Differences between multiple groups were analyzed by analysis of variance (ANOVA), whereas differences between the two groups were analyzed using a *t* test. A value of *P* < 0.05 was considered to be statistically significant.

## Results

### Treatment of BMSCs with 50 μM H_2_O_2_ significantly enhanced cell viability, proliferation, and migration by augmenting the SDF-1/CXCR4/CXCR7 axis

**Fig. 1:**
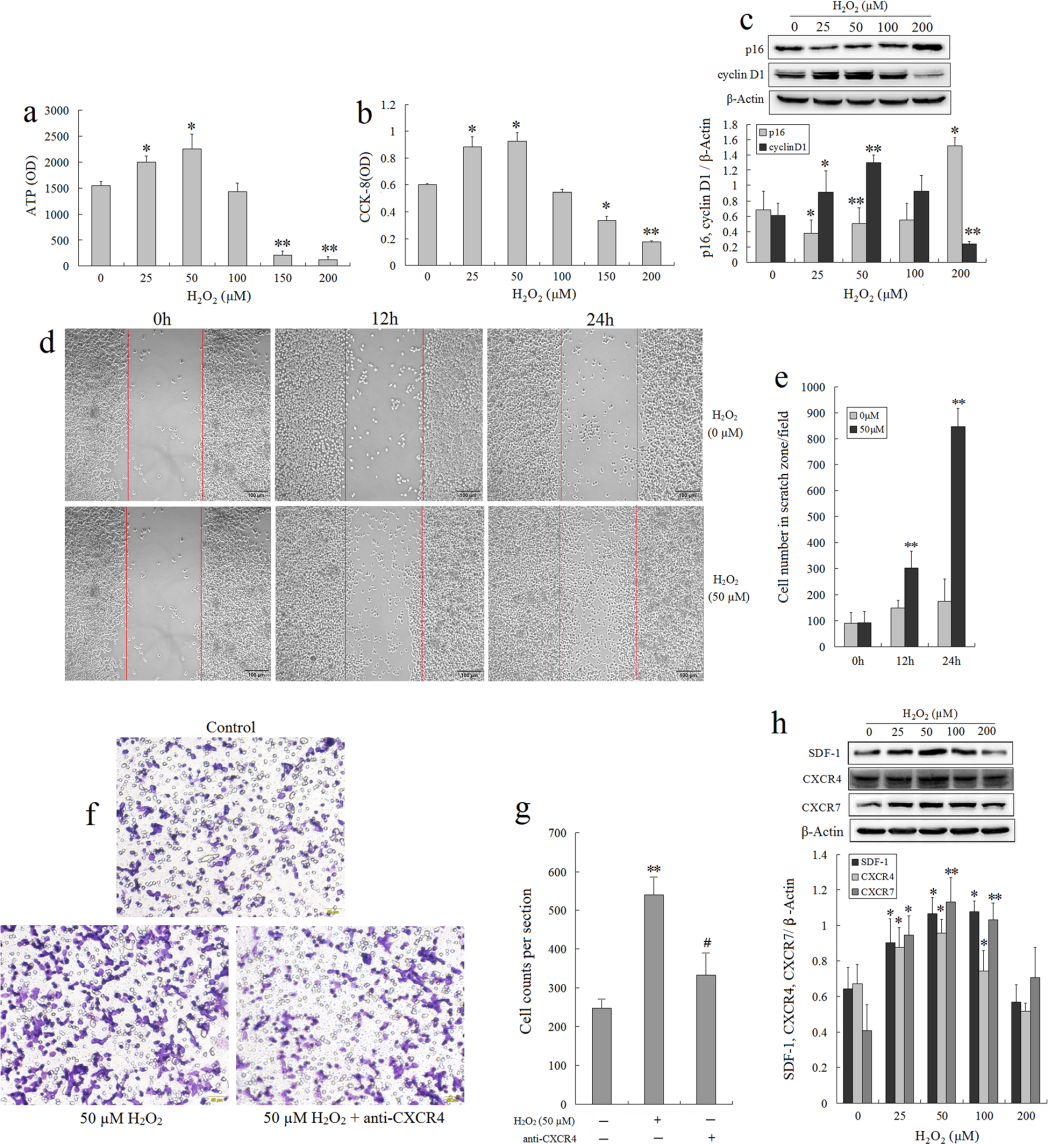
Optimization of H_2_O_2_ concentrations for preconditioning BMSCs. **a** BMSCs were treated with the indicated concentrations of H_2_O_2_ (0–200 μM) for 12 h. The cell viability was measured by ATP assay. Values represent the mean ± standard errors of the means (SEM) (*n* = 3). **b** The effect of H_2_O_2_ on BMSC proliferation determined by the CCK-8 assay. H_2_O_2_ at 25 and 50 μM significantly promoted the proliferation of BMSCs compared with the control (*n* = 3). **c** Western blot analysis of cyclin D1 and p16 expressions in BMSCs exposed to different low concentrations H_2_O_2_ for 12 h. Values represent the mean ± SEM (*n* = 3). *P < 0.05, **P < 0.01 vs. 0 μM H_2_O_2_. **d** Representative images of the scratch assay showed the migration of BMSCs with or without 50 μM of H_2_O_2_ treatment for 12 h and 24 h. Scale bar, 100 μm. **e** Quantification of the number of cells which have migrated into the scratch zone; 50 μM of H_2_O_2_-treated cells had a significantly increased migration compared with control untreated cells (*n* = 3). **f** Images of transmigrated BMSCs (stained with crystal violet) are shown (× 200 magnification). Scale bar, 100 μm. **g** Quantitative analysis of migrated cells. Data is presented as mean ± SEM of the six independent experiments (*n* = 6). **h** Western blot analysis of SDF-1 and CXCR4 expressions in BMSCs exposed to different low doses of H_2_O_2_ for 12 h. Values represent the mean ± SEM (*n* = 3). *P < 0.05, **P < 0.01 vs. the control group (0 μM H_2_O_2_); ^#^*P* < 0.05 vs. the 50 μM H_2_O_2_ group

To define the optimal concentration of H_2_O_2_ in preconditioning BMSCs, we selected five different concentrations between 25 and 200 μM based on previous reports [29] and investigated its effects on cellular properties of viability, proliferation, migration, and differentiation. By using the ATP assay and CCK-8 assay, we found that H_2_O_2_ at 25 μM and 50 μM significantly increased the viability of BMSCs (Fig. 1a) and promoted the proliferation of BMSCs (Fig. 1b), accompanied by an increase in cyclin D1 expression and decrease in p16 expression, whereas 150 and 200 μM H_2_O_2_ showed a significant inhibitory effect compared with that of the control. Fifty-micromolar H_2_O_2_ caused a peak cyclin D1 expression and a low p16 expression, which represented the optimal concentration of H_2_O_2_ for BMSC preconditioning in terms of cell proliferation/viability from this study (Fig. 1c).

Next, the effect of 50 μM H_2_O_2_ on the migration ability of BMSCs was evaluated using a scratch assay. The results showed that 50 μM H_2_O_2_ treated enhanced cell migration significantly compared with control cells untreated with H_2_O_2_ (Fig. 1d, e). The Boyden chamber assay was further demonstrated that the number of migrating BMSCs in the H_2_O_2_ group was much more than in that of the control group; however, the enhanced migration capacity was attenuated by pretreatment with anti-CXCR4 antibody (Fig. 1f, g). Simultaneously, we found that the expression of SDF-1, CXCR4, and CXCR7 in the H_2_O_2_ group was significantly upregulated at 25 μM and 50 μM compared to that of the control (Fig. 1h). These results suggested that the 50 μM H_2_O_2_ treatment for 12 h could significantly enhance the BMSC viability, proliferation, and migration capabilities, which may be through activating the SDF-1/CXCR4/CXCR7 pathway.

Additionally, we found that the BMSCs still have maintained the multipotent differentiation capacity of stem cells even after pretreated with 50 μM H_2_O_2_ for 12 h (Supplement Fig. 1). They could differentiate into adipocytic (Oil red O staining), chondrocytic (Toluidine blue, Alcian blue), and osteoblastic (alkaline phosphatase) lineages. These results showed that 50 μM H_2_O_2_ treatment did not affect the differentiation potential of BMSCs.

### Optimal H_2_O_2_ preconditioning suppressed cell death of BMSCs via the mitochondria-mediated mechanism

**Fig. 2:**
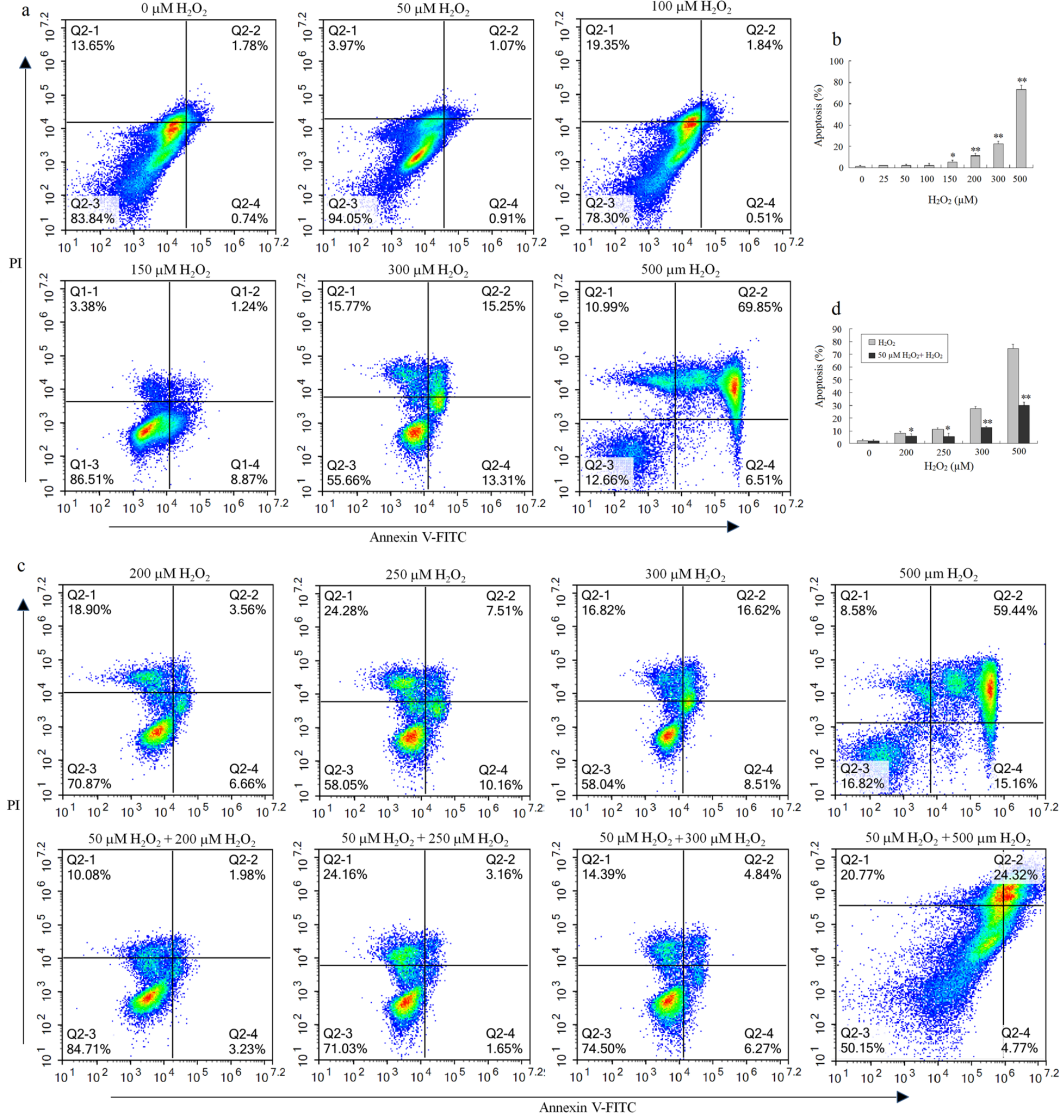
Optimal H_2_O_2_ preconditioning provides anti-apoptotic protection to BMSCs. **a** Representative images of flow cytometric analysis by annexin V-FITC/PI dual staining after BMSCs treated for 24 h with various concentrations of H_2_O_2_ (25, 50, 100, 150, 200, 300, and 500 μM). The bottom right quadrant represents annexin V-FITC-stained cells (early-phase apoptotic cells), and the top right quadrant represents PI- and annexin V-FITC-dual-stained cells (late-phase apoptotic/necrotic cells). **b** Apoptotic cells are represented as the percentage of annexin V single-positive plus annexin V-FITC/PI double-positive cells (*n* = 3). **c** Representative images of flow cytometric analysis by annexin V-FITC/PI dual staining. Confluent BMSCs were pretreated with or without 50 μM of H_2_O_2_ for 12 h. After removing the supernatants, cells were incubated with fresh medium in the presence of various concentrations of H_2_O_2_ (200, 250, 300, and 500 μM) for an additional 12 h. **d** Apoptotic cells are represented as the percentage of annexin V single-positive plus annexin V-FITC/PI double-positive cells (*n* = 3). *P < 0.05, **P < 0.01 vs. the control groups (0 μM H_2_O_2_) or the H_2_O_2_ un-pretreatment group

Based on the above results, further, the protective effect of 50 μM H_2_O_2_ preconditioning for 12 h on BMSCs against oxidative stress-induced apoptosis was detected by the flow cytometric (FCM) analysis. The results showed that exposure to 25–100 μM H_2_O_2_ did not cause BMSC apoptosis (Fig. 2a). However, exposure to 150–500 μM H_2_O_2_ induced an obvious increase in the apoptotic rate of BMSCs in a dose-dependent manner (Fig. 2a, b), compared with the control. The results showed that H_2_O_2_ exceeding 100 μM might induce oxidative injury thereby leading to cell apoptosis. However, the apoptotic rate of BMSCs was very significantly lower in 50 μM H_2_O_2_-preconditioned each group than in corresponding high doses of H_2_O_2_ groups (Fig. 2c, d). This result suggested that 50 μM H_2_O_2_ preconditioning for 12 h remarkably attenuated high concentration H_2_O_2_-induced apoptosis in BMSCs.

**Fig. 3:**
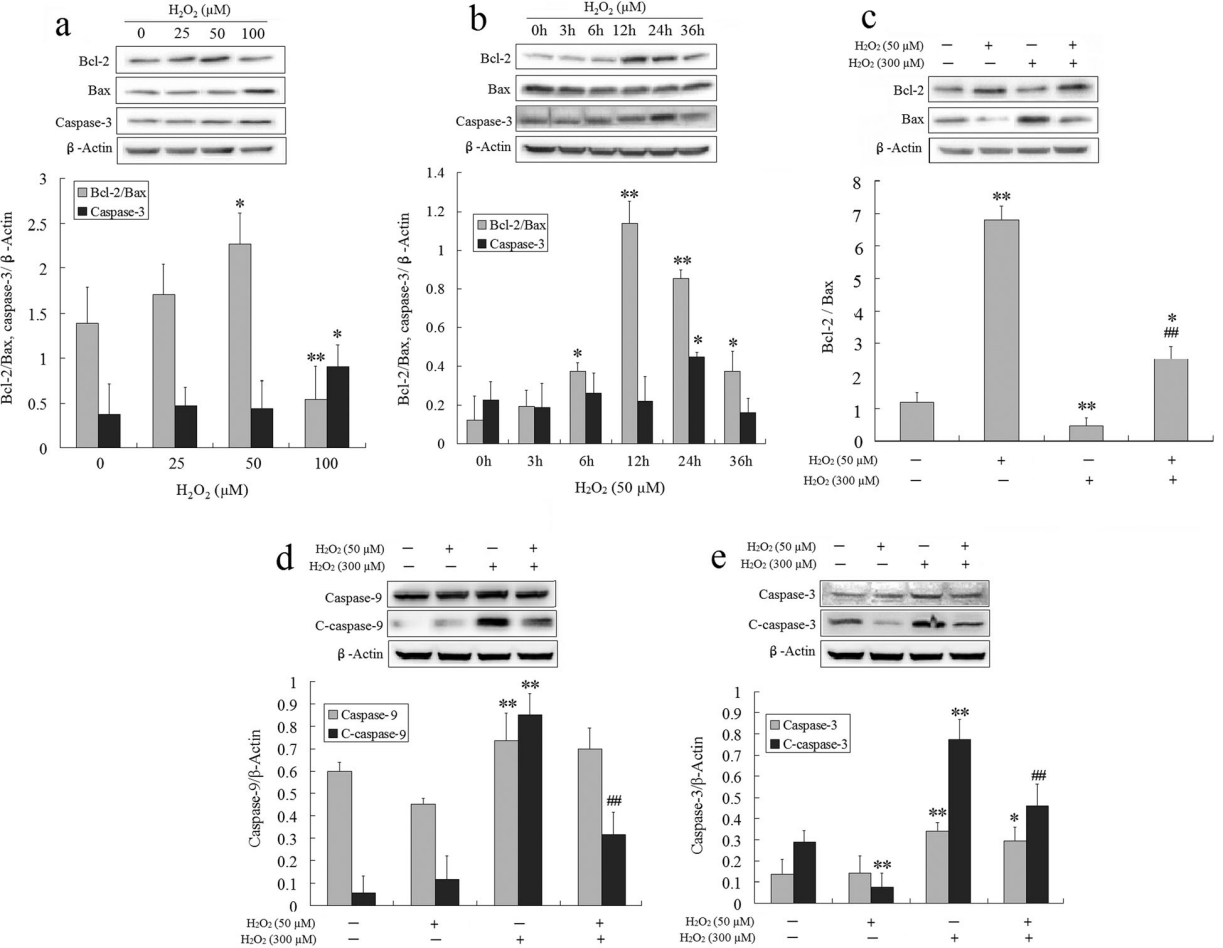
Effect of optimal H_2_O_2_ on the mitochondria-mediated apoptosis pathway in BMSCs. Western blot analysis of the expressions of Bcl-2, Bax, and caspase-3 proteins in BMSCs exposed to various low concentrations of H_2_O_2_ (25, 50, and 100 μM) for 12 h. **a** The bar graphs showed the ratio of Bax/Bcl-2 and the quantification of the caspase-3 protein in BMSCs. **b** Western blot analysis of the expressions of Bcl-2, Bax, and caspase-3 in BMSCs exposed to 50 μM H_2_O_2_ for different time intervals (3, 6, 12, 24, and 36 h). The bar graphs showed the ratio of Bax/Bcl-2 and the quantification of the caspase-3 protein; Western blot analysis of the expressions of Bcl-2, Bax, caspase-9/3, and cleaved caspase-9/3 in MSCs exposed to 300 μM H_2_O_2_ for 24 h after pretreatment with 50 μM H_2_O_2_ for 12 h. **c** The bar graphs showed the ratio of Bax/Bcl-2 in BMSCs. **d** The bar graphs showed the quantification of the caspase-9 and cleaved caspase-9 protein. **e** The bar graphs showed the quantification of the caspase-3 and cleaved caspase-3 protein. All results are presented as mean ± SEM (*n* = 3). *P < 0.05, **P < 0.01 vs. the control group; ^##^*P* < 0.01 vs. the 300 μM H_2_O_2_ group

Oxidative stress induces apoptosis through the intrinsic mitochondrial pathway [30]. The ratio of anti-apoptotic Bcl-2 and pro-apoptotic Bax (Bcl-2/Bax) is the key to the regulation of the mitochondrial apoptotic pathway [30]. Treatment of BMSCs with 50 μM H_2_O_2_ for 12 h resulted in a peak Bcl-2/Bax expression ratio compared to that of the control, but Bax and caspase-3 were not affected (Fig. 3a). Subsequently, after treatment of BMSCs with 50 μM H_2_O_2_ for different time intervals (3, 6, 12, 24, and 36 h), it was found that when H_2_O_2_ treated for 12 h, the Bcl-2/Bax ratio reached a peak, while the expression of Bax and caspase-3 was not affected; however, the two pro-apoptosis proteins were increased when the action time exceeds 12 h (Fig. 3b). The data also confirmed that treatment with 50 μM H_2_O_2_ for 12 h might be the optimal treatment condition for BMSCs preconditioning in vitro in terms of the expression ratio of anti- and pro-apoptotic proteins.

Further, the expressions of apoptosis-related proteins in BMSCs were measured by Western blot after cells were pretreated with 50 μM H_2_O_2_ for 12 h, and then treated with or without 300 μM for an additional 24 h. As shown in Fig. 3c–e, 300 μM H_2_O_2_ led to a sharply decreased Bcl-2/Bax expression ratio (Fig. 3c) and increased expressions of caspase-3 and caspase-9, and cleaved caspase-3 and cleaved caspase-9 proteins (Fig. 3d, e), while 50 μM H_2_O_2_ preconditioning significantly increased the Bcl-2/Bax expression ratio, and decreased the levels of caspase-3, cleaved caspase-3, and cleaved caspase-9 without having effects on caspase-9. These results indicated that preconditioning BMSCs with 50 μM H_2_O_2_ for 12 h could markedly suppress oxidative stress-induced activation of the mitochondrial apoptosis pathway through inhibiting the expression of pro-apoptotic proteins.

### Fifty-micromolar H_2_O_2_ preconditioning attenuated oxidative stress-induced mitochondrial dysfunction by inhibiting mitochondrial reactive oxygen species (mtROS) generation in BMSCs

**Fig. 4:**
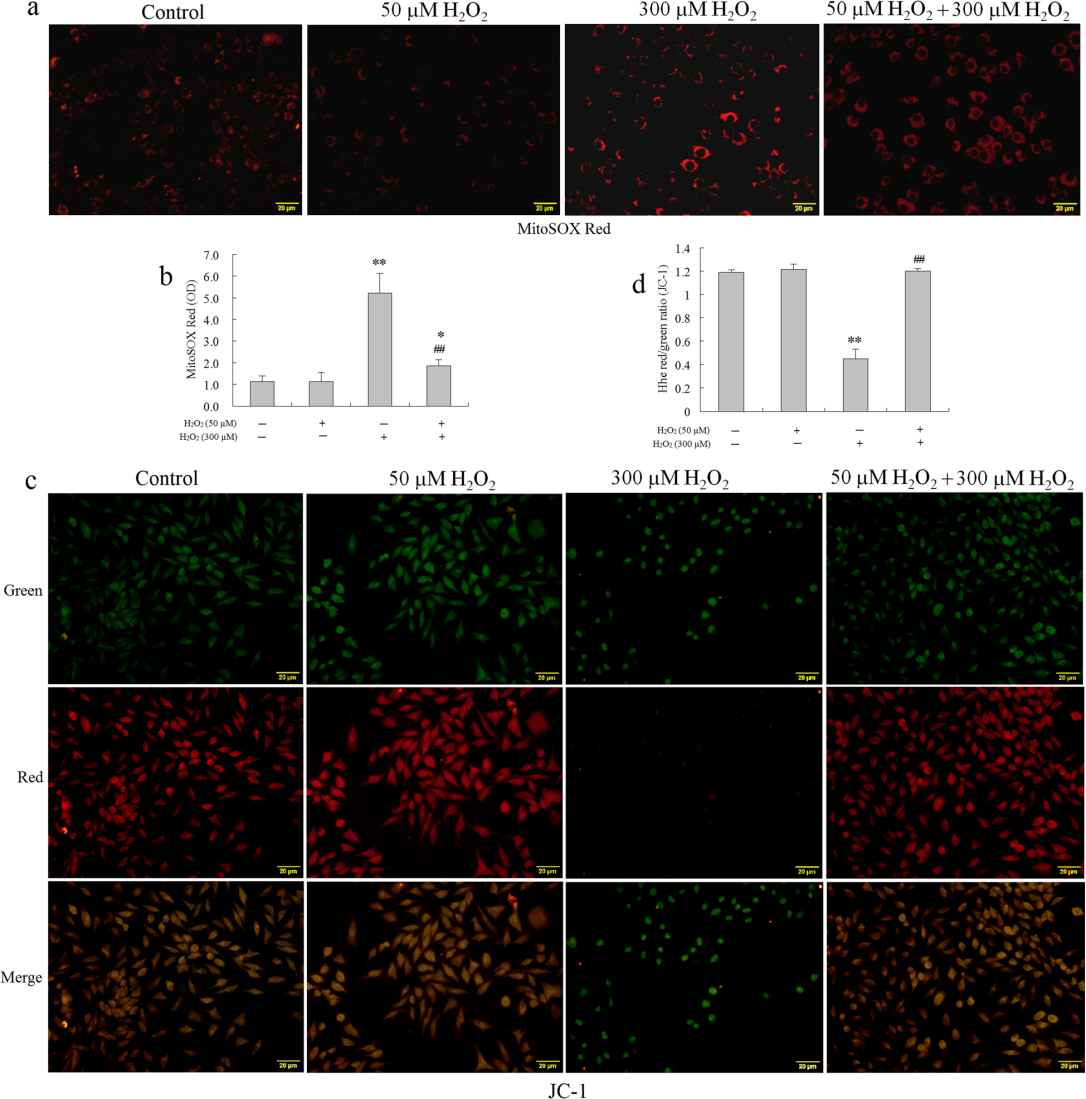
Fifty-micromolar H_2_O_2_ preconditioning improved the redox state of the mitochondria; 50 μM H_2_O_2_ attenuated oxidative stress (300 μM)-induced mitochondrial dysfunction by inhibiting mtROS generation in BMSCs. Cells were pretreated with 50 μM H_2_O_2_ for 12 h, washed, and then incubated with fresh medium in the presence or absence of 300 μM H_2_O_2_ for an additional 24 h. **a** The mitochondrial O_2_^•**−**^ production was determined using MitoSOX Red staining and observed under the inverted fluorescence microscopy. Scale bar, 20 μm. **b** The mitochondrial O_2_^•**−**^ levels were measured using MitoSOX Red staining, and the fluorescence values were read at an excitation wavelength of 510 nm and emission wavelength of 579 nm (*n* = 4). The absorbance values were expressed as the mitochondrial O_2_^•−^ levels. **c** Fifty-micromolar H_2_O_2_ suppressed oxidative stress-induced collapse of mitochondrial membrane potential in BMSCs. Changes of Δ*ψm* was observed using the inverted fluorescence microscopy. Red fluorescence was emitted by JC-1 aggregates in healthy mitochondria with polarized inner mitochondrial membranes, whereas green fluorescence was emitted by cytosolic JC-1 monomers, indicating Δ*ψm* dissipation. Scale bar, 20 μm. Merged images indicate the co-localization of JC-1 aggregates and monomers. **d** Δ*ψm* in each group was calculated as the ratio of red to green fluorescence, indicating changes in mitochondrial membrane potential. All results are presented as mean ± SEM of at least four independent experiments (*n* = 4). *P < 0.05, **P < 0.01 vs. the control group; ^##^*P* < 0.01, vs. the 300 μM H_2_O_2_ group

Accumulating excessive ROS in the mitochondria is considered to be one of the major causes of oxidative stress in cells, resulting in perturbation of mitochondrial redox homeostasis and mitochondrial dysfunction. Mitochondrial reactive oxygen species (mtROS) homeostasis plays an essential role in preventing oxidative injury in BMSCs [31]. Since mitochondria are the main source of ROS, determining the fate of cells through regulating multiple signal transduction pathways, and mitochondria are also vulnerable targets. The protective effect of 50 μM H_2_O_2_ preconditioning BMSCs on the regulation of mitochondrial redox homeostasis (mitochondrial redox status) was evaluated using MitoSOX Red, a highly selective fluorescent probe for detection of the O_2_^•−^ generated within the mitochondria. The MitoSOX Red reagent is a live-cell permeant and is selectively targeted to the mitochondria. Once in the mitochondria, MitoSOX Red is oxidized by O_2_^•−^ and exhibits red fluorescence [32]. As shown in Fig. 4a, b, 300 μM H_2_O_2_ significantly increased mitochondrial O_2_^•−^ generation compared with the control group. However, this effect was dramatically mitigated by 50 μM H_2_O_2_ preconditioning in BMSCs. Since the mitochondrial membrane potential (Δ*ψm*) is significantly affected by the mitochondrial redox status, further, the effect of 50 μM H_2_O_2_ preconditioning on mitochondrial membrane potential (Δψm) in BMSCs was measured using JC-1 probe. JC-1 can selectively enter the mitochondria and reversibly changes color as the Δ*ψm* changes [33]. As shown in Fig. 4b, c, there was a significantly increased green fluorescence in cells exposed to 300 μM H_2_O_2_, suggesting that the mitochondrial membrane was depolarized. However, 50 μM H_2_O_2_ pretreatment repressed the changes of Δ*ψm* induced by 300 μM H_2_O_2_, indicated by a reduction of green fluorescence and restoration of red fluorescence. Moreover, These results suggested that 50 μM H_2_O_2_ preconditioning for 12 h remarkably suppressed oxidative stress-induced collapse of mitochondrial membrane potential (Δ*ψm*) in BMSCs, which was further confirmed that 50 μM H_2_O_2_ preconditioning protected against oxidative stress-induced apoptosis in BMSCS, at least partly, via restoring MMP function.

### Optimal H_2_O_2_ preconditioning activated PI3K/Akt/mTOR signaling while inhibited GSK-3β activity in BMSCs

**Fig. 5:**
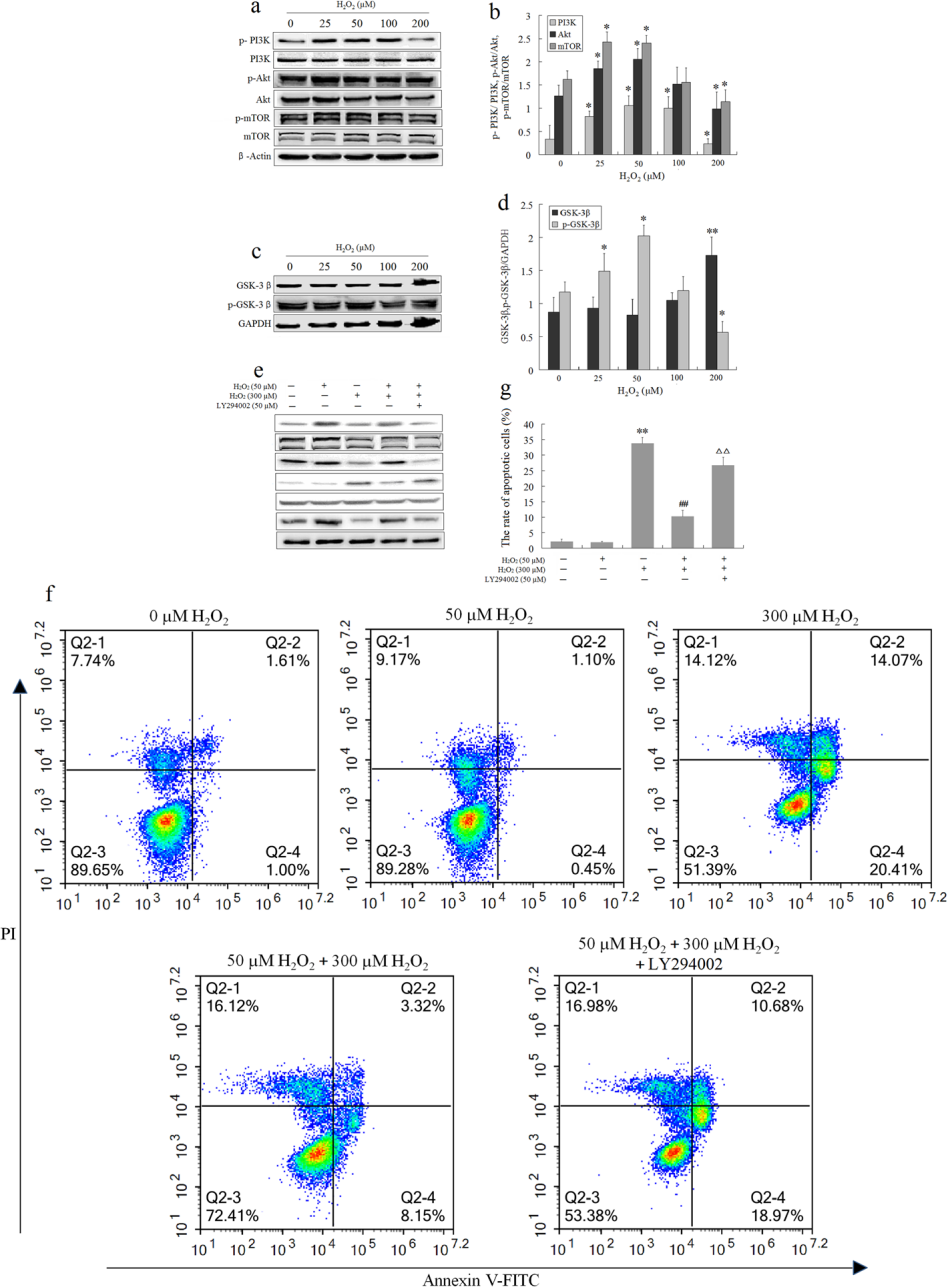
The protective effect of optimal H_2_O_2_ preconditioning on the activation of PI3K/Akt/mTOR/GSK-3β signaling. **a**, **c** Western blot analysis of the expressions of total and phosphorylated PI3K, Akt, mTOR, and GSK-3β proteins in BMSCs exposed to various concentrations of H_2_O_2_ (25, 50, 100, and 200 μM) for 24 h. **b** The bar graphs show the quantitation of phosphorylation of PI3K, Akt, and mTOR protein. **d** The bar graphs show the quantitation of phosphorylation of GSK-3β protein. Values represent the mean ± SEM (*n* = 3). *P < 0.05, **P < 0.01 vs. the control group. **e** Western blot analysis of the expressions of phosphorylated Akt, mTOR, and GSK-3β proteins, and apoptosis-related protein Bcl-2 and cleaved caspase-3 proteins in H_2_O_2_-preconditioned BMSCs exposed to 300 μM for 24 h after 2 h pre-incubation with 50 μM LY294002. **f** Representative images of flow cytometric analysis by annexin V-FITC/PI dual staining. **g** Apoptotic cells are represented as the percentage of annexin V single-positive plus annexin V-FITC/PI double-positive cells. Values represent the mean ± SEM (*n* = 3). **P < 0.01 vs. the control group; ^##^*P* < 0.01 vs. the 300 μM H_2_O_2_ group; ^ΔΔ^*P* < 0.05 vs. the H_2_O_2_-preconditioned group

The PI3K/Akt/mTOR pathway and GSK-3β protein play a vital role in promoting stem cell survival in response to oxidative stress [17–19, 29]. Exposure to 25–50 μM H_2_O_2_ induced an obvious increase in the phosphorylation levels of PI3K, Akt, mTOR, and GSK-3β in BMSCs, but they were significantly decreased at 200 μM H_2_O_2_; inversely, the GSK-3β expression was upregulated at higher H_2_O_2_ (Fig. 5a–d). The results indicated that low doses of H_2_O_2_ could activate the PI3K/Akt/mTOR pathway and inhibit GSK-3β activity in BMSCs, whereas a high dose of H_2_O_2_-induced (200 μM) oxidative stress inhibited this pathway and enhanced GSK-3β activity. Further, the results showed that 50 μM H_2_O_2_ preconditioning could largely prevent 300 μM H_2_O_2_-induced inactivating PI3K/Akt/mTOR pathway and activating GSK-3β. This effect was significantly blocked by PI3K inhibitor LY294002 (Fig. 5e); simultaneously, the protective effect of 50 μM H_2_O_2_ preconditioning was counteracted by LY294002 with a remarkable increase in the apoptotic rate of BMSCs (Fig. 5f, g). These results showed that the protective effect of 50 μM H_2_O_2_ against oxidative stress-induced BMSC apoptosis was mediated, at least in part, by the activation of the PI3K/Akt/mTOR pathway and inhibition of GSK-3β activity.

### Optimal H_2_O_2_ preconditioning enhanced BMSCs’ tissue engraftment and wound healing in mice

**Fig. 6:**
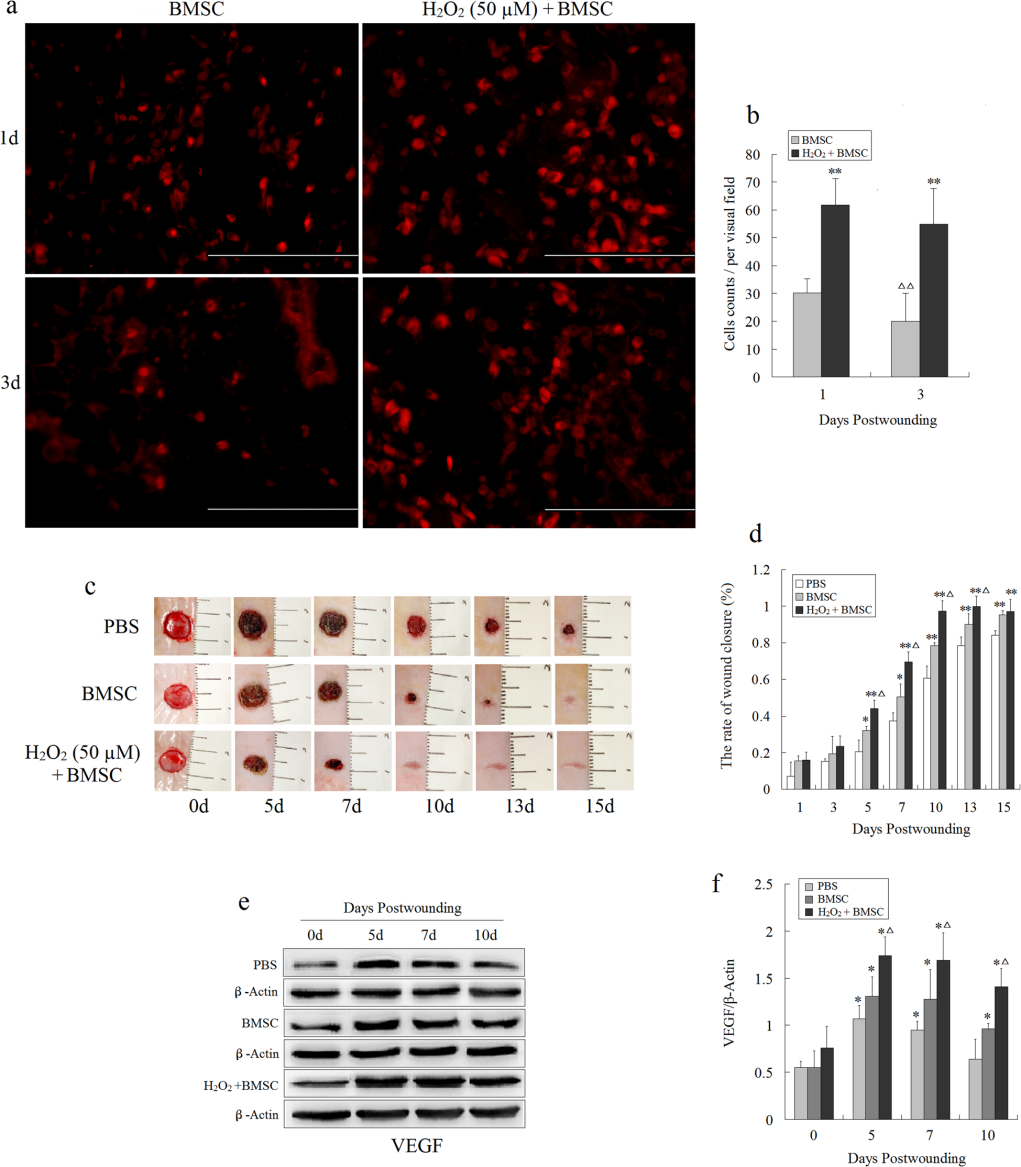
Optimal H_2_O_2_ preconditioning enhances BMSC tissue engraftment and would closure. The full-thickness wounds were created at the dorsum of mice, followed by the tail vein injection of PBS and DiI-labeled BMSCs with or without H_2_O_2_ preconditioning for 12 h, respectively. **a**, **b** Histological analysis of the number of transplanted DiI-labeled BMSCs (red) migrating into newly formed granulation tissue at days 1 and 3 post-wounding. Scale bar, 100 μm. Data are presented as the total cells per field (× 200) ± SEM (*n* = 5). **P < 0.01 vs. the BMSCs group; ^ΔΔ^*P* < 0.01 vs. the day 1 post-wounding within the same group. **c** Gross view of excisional wounds at days 0, 5, 7, 10, 13, and 15 post-wounding. **d** The rate of wound closure after BMSCs engrafting into the wounds. Data are shown as means ± SEM (*n* = 5). **e**, **f** Western blot analysis of the expressions of VEGF of wound tissue after transplantation of BMSCs. Data are shown as means ± SEM (*n* = 3). *P < 0.05 vs. the PBS group; ^Δ^*P* < 0.05 vs. the BMSCs group

In order to further confirm whether optimal H_2_O_2_ preconditioning enhanced the target homing efficiency of tail vein-infused BMSCs toward wounds and improved the therapeutic potential of BMSCs in wounds, full-thickness wounds were created at the dorsum of Balb/C mice, followed by the tail vein injection of DiI-labeled BMSCs. The number of DiI-labeled BMSCs in the H_2_O_2_-preconditioned group at 1 day and 3 days post-wounding significantly increased compared to that of the un-preconditioned BMSCs group. Compared with the first day post-wounding, the number of BMSCs in the H_2_O_2_-preconditioned group decreased only slightly on the third day; however, the number of BMSCs in the un-preconditioned BMSCs group was significantly reduced on the third day (Fig. 6a, b). The result hinted that the H_2_O_2_ preconditioning could not only increase BMSCs migrating and homing toward wounds, but also prolong the residence time of transplanted BMSCs. In addition, the results showed that the original wounds in the H_2_O_2_-preconditioning group were significantly smaller compared to the PBS control and BMSC alone groups at days 5, 7, 10, 13, and 15 post-wounding (Fig. 6c) and were completely healed at day 10 post-wounding, but none of the other two groups. Correspondingly, the wound closure rate was the highest in the H_2_O_2_-preconditioned group from day 5 to day 15 post-wounding (Fig. 6d). Therefore, the optimal H_2_O_2_ preconditioning not only increased the migration of BMSCs into the wound areas, but also accelerated wound closure.

**Fig. 7:**
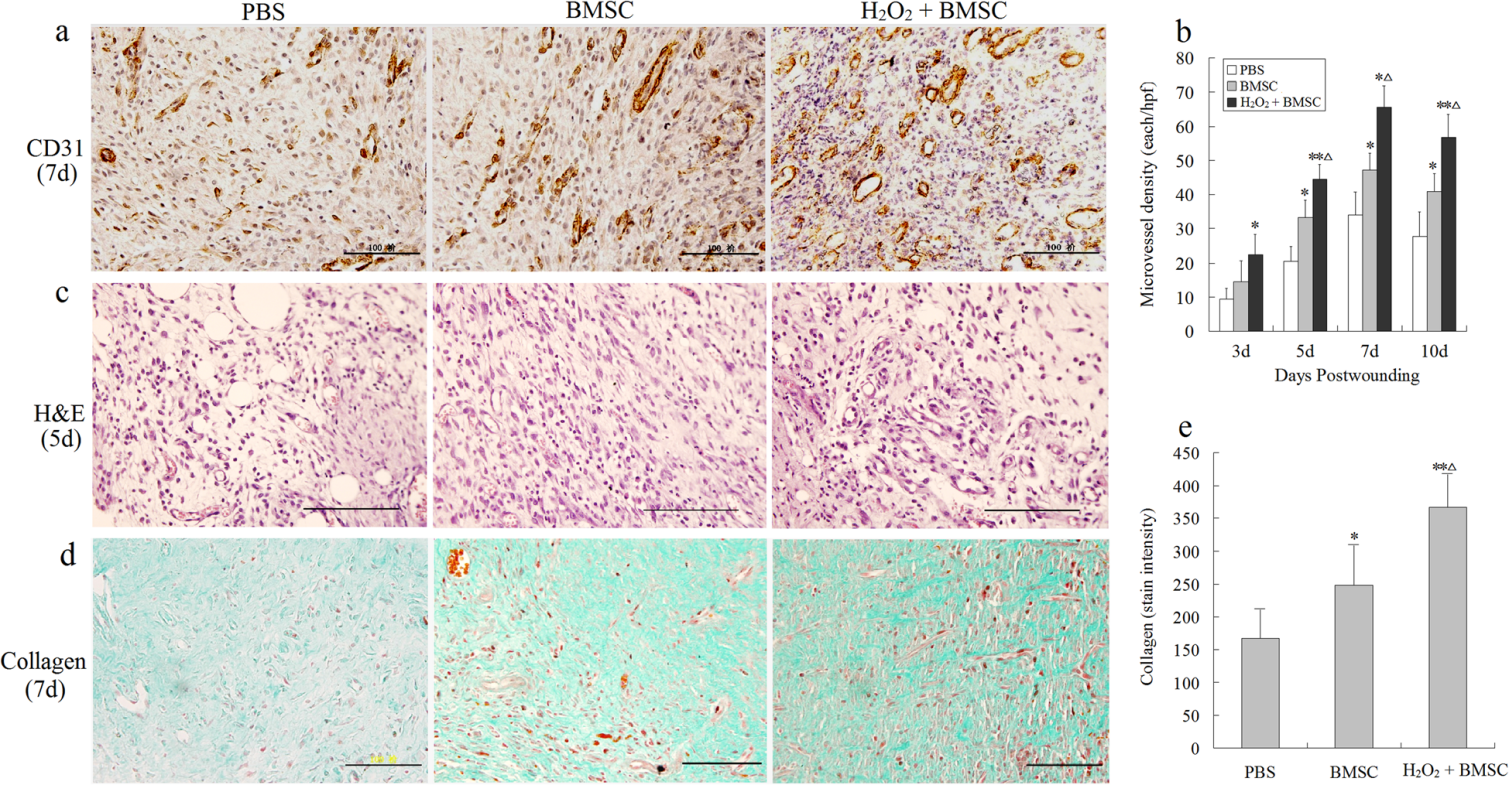
Effect of optimal H_2_O_2_-preconditioned BMSCs on wound healing in mice. **a** Representative images of mouse CD31 staining of wounds in PBS, BMSC, and H_2_O_2_-preconditioned groups. Scale bar, 50 μm. **b** Microvessel density in the wounds was assessed by CD31-positive staining for microvessels, expressed as the number of CD31-positive microvessels per field. **c** H&E staining of wound granulation tissue in PBS, BMSC, and H_2_O_2_-preconditioned BMSCs groups at 5 days post-wounding. Scale bar, 50 μm. **d** Evaluation of collagen deposition by Masson staining at day 7 post-wounding. Representative × 40 image from the center of the granulation tissue of each group displaying cells and granulation within the wound center. Scale bar, 50 μm. **e** Qualification of the stain intensity of blue collagen shown in **d**. Data are shown as means ± SEM (*n* = 3). *P < 0.05 vs. the PBS group; ^Δ^*P* < 0.05 vs. the BMSCs group

Further, the growth factor detection and histological analysis of wounds were performed to assess the degree of wound healing and regeneration. The results showed that the level of VEGF in the two BMSCs groups was significantly higher compared to that of the PBS group (Fig. 6e, f), and the microvessel density was the highest in the H_2_O_2_-preconditioned group at days 5, 7, and 10 post-wounding (Fig. 7a, b). Similarly, compared with the other two groups, H&E staining showed the greatest increase in cell density and blood vessel density in the granulation tissue of the H_2_O_2_-preconditioned group at days 5 post-wounding (Fig. 7c). Moreover, a larger and better-organized collagen deposition was observed in the wounds of H_2_O_2_-preconditioned BMSC mice at days 7 post-wounding, showing a significantly higher collagen density compared with the PBS control and BMSCs alone groups (Fig. 7d, e). These data indicated that the transplanted BMSCs after optimal H_2_O_2_-preconditioning not only augmented tissue engraftments and accelerated wound closure, but also significantly promoted collagen deposition and angiogenesis in wounds, and thus facilitated would healing.

## Discussion

Although cell-based therapy plays an important role in the field of skin tissue regeneration, low engraftment efficiency and poor survival rate of transplanted stem cells triggered by oxidative stress at the wound tissue limit the therapeutic potential of MSCs [2, 3]. Accumulating evidences suggest that H_2_O_2_ preconditioning could enhance the migration and survival of MSCs [22–24, 34]. However, optimal H_2_O_2_ concentration, suitable treatment time course, and the underlying mechanism remained largely undetermined.

Different concentrations of H_2_O_2_ profoundly affect the biological functions of stem cells [25–27]. For a long time, H_2_O_2_ was considered deleterious molecules. However, recent studies suggest that H_2_O_2_ is necessary for effective self-renewal in stem cells [16, 25, 35]. In general, moderate levels of H_2_O_2_ may function as signals to promote cell proliferation and survival, playing a growth factor-like role in cells. However, a severe increase of H_2_O_2_ induces senescence and oxidative stress in MSCs [16, 27]. Thus, exploring the optimal concentration of H_2_O_2_ for MSC preconditioning in vitro is a prerequisite for improving the therapeutic potential of MSCs. Indeed, during the oxidative stress experiment of BMSCs with various concentrations of H_2_O_2_, we identified that H_2_O_2_ at 25 μM and 50 μM significantly facilitated the proliferation of BMSCs, accompanied by an increase in the expression of the cell cycle-promoting protein cyclin D1 and decrease in the cell cycle blocker p16 expression; 100 μM H_2_O_2_ has not impacted cell growth, and over 150 μM H_2_O_2_ induced cell growth arrest with downregulating cyclin D1 and upregulating p16 expressions, accompanied by BMSC apoptosis. Importantly, we found 50 μM H_2_O_2_ optimally promoted BMSC proliferation and maximized the expression of cyclin D1, while the greatest degree of suppression of p16 expression, which was suggested 50 μM H_2_O_2_, may be an optimal concentration to promote stem cell growth. Simultaneously, we confirmed that 50 μM H_2_O_2_ maximized the promotion of SDF-1 and its receptors CXCR4/7 expression that were conducive to the enhancement of cell migration and survival, respectively [7, 8]. In fact, we observed that 50 μM H_2_O_2_ treatment could markedly promote the migration capability of BMSCs in vitro; however, the effect was eliminated by the CXCR4 antibody, which indicated H_2_O_2_-promoting migration of BMSCs through augmenting the SDF-1/CXCR4 axis. Thus, these results confirmed the important effect of H_2_O_2_ concentration on the biological characteristics of stem cells and also confirmed that a low concentration of H_2_O_2_ had a growth factor-like effect to promote stem cell proliferation. Although it has been reported that the concentration of stem cells preconditioned with H_2_O_2_ is 25 μM, 50 μM, and 100 μM [23, 24], there is no sufficient basis for deciding which H_2_O_2_ concentration to be chosen, and we believed that 50 μM H_2_O_2_ represent the optimal concentration of MSC preconditioning.

It is well known that oxidative stress induces apoptosis through the intrinsic mitochondrial pathway [29]. The expression ratio of anti-apoptotic Bcl-2 and pro-apoptotic Bax (Bcl-2/Bax) is the key to maintaining mitochondrial membrane stability, preventing mitochondrial depolarization and the release of cytochrome c into the cytoplasm, and thereby inhibiting the mitochondrial apoptotic pathway [29]. High concentrations of H_2_O_2_-induced oxidative stress have been shown to induce mitochondrial injury by reducing mitochondrial membrane potential and decreasing the expression ratio of Bcl-2/Bax, subsequently initiating apoptosis [30]. In the present study, we similarly confirmed that 50 μM H_2_O_2_ could maximize the upregulation of Bcl-2 and Bcl-2/Bax expression ratio with no changes in pro-apoptotic proteins Bax and cleaved-caspase3 expressions, when using different concentrations of H_2_O_2_ to stimulate stem cells for 12 h. Further, it was found that pretreatment BMSCs for 12 h was the optimal time course when using 50 μM H_2_O_2_ to treat BMSCs with different time course, because the Bcl-2 and Bcl-2/Bax expression ratio peaked with no changes in Bax and cleaved-caspase3 expressions. Lastly, we suggested that BMSC preconditioning with 50 μM H_2_O_2_ for 12 h is the optimal protocol for resistance to oxidative stress apoptosis through maximizing increasing the Bcl-2/Bax expression ratio. In addition, we also confirmed that 50 μM H_2_O_2_ could markedly promote the migration capability of BMSCs through maximizing augmenting the SDF-1/CXCR4/7 axis. Further, the results confirmed that the optimal preconditioning protocol (50 μM H_2_O_2_ for 12 h) could significantly enhance BMSC survival under oxidative stress and suppress the mitochondrial apoptotic pathway through increasing the expression ratio of Bcl-2/Bax and inhibition of apoptosis executive proteins cleaved caspase-9 and cleaved caspase-3 expressions. However, LY294002 abolished the cytoprotective effect of 50 μM H_2_O_2_ preconditioning. The data suggested that the PI3K/Akt pathway was involved in the preconditioning-induced cytoprotective effect.

**Fig. 8:**
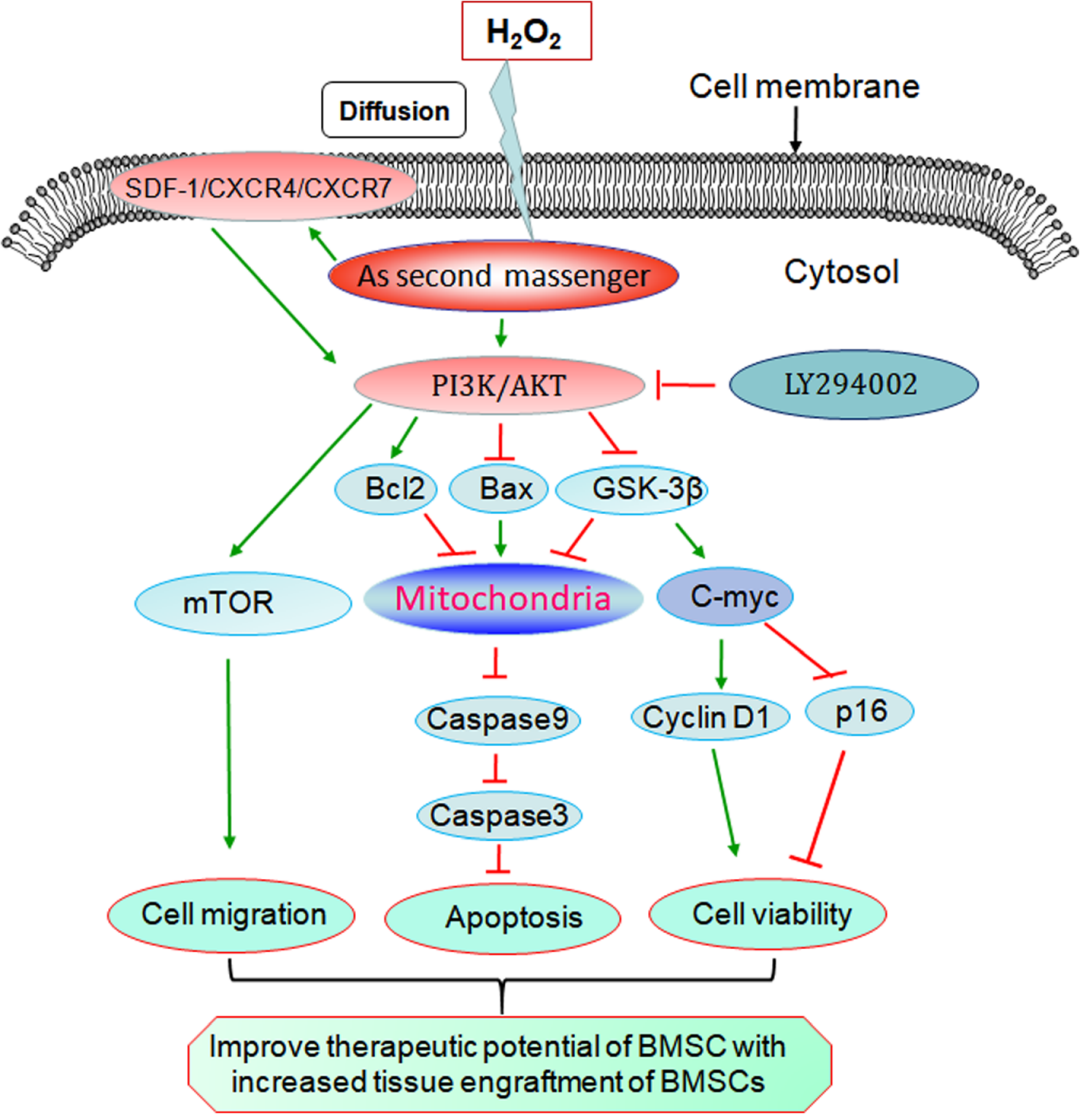
Schematical representation of the protective mechanism of H_2_O_2_ preconditioning for improving the therapeutic potential of BMSCs. A low dose of H_2_O_2_ (50 μM) acts in the signaling pathways as a second messenger. H_2_O_2_ preconditioning-induced BMSC protection seems to involve augmenting the SDF-1/CXCR4 axis and activation of PI3K/Akt/mTOR and inhibiting GSK-3β activity, leading to enhancing abilities of BMSC proliferation, migration, angiogenesis, and anti-apoptosis. And the PI3K/Akt pathway is also one of the downstream pathways that activated the SDF-1/CXCR4 axis [9, 10, 18–21]. In addition, low-dose H_2_O_2_ directly activated this pathway through diffusion into the cytoplasm as a second messenger may be another activation pathway [17, 25]. The PI3K inhibitor (LY294002) could block the protective effect induced by 50 μM H_2_O_2_ preconditioning

It was reported that the PI3K/Akt pathway has been shown to play a major role in the promotion of cell survival, and prevention of apoptosis in response to oxidative stress [16, 21, 36]. The activation of PI3K/Akt causes a cascade of downstream responses from mTOR, Bcl-2, Bax, and GSK-3β, which all regulate cellular functions, such as cell migration, growth, proliferation, survival, and differentiation [16, 21] (Fig. 8). The PI3K/Akt pathway regulates cell migration via activation of mTOR, promotes cell survival via Bcl-2/Bax, and facilitates cell proliferation, differentiation, and resistance apoptosis via inhibition of GSK-3β [37, 38]. Akt directly phosphorylates GSK-3β at Ser9 which negatively regulates its kinase activity [39]. Phosphorylated GSK-3β, a key molecule, inhibits oxidative stress-induced apoptosis through inhibition of the opening of mitochondrial permeability transition pore (mPTP), thereby suppressing cytochrome C release for mitochondria to cytosol and preventing cell apoptosis [39]. And also, Akt-induced GSK-3β phosphorylated leads to the accumulation of β-catenin in the nucleus [38], which is a transcriptional factor that increases the expression of c-Myc to promote cell proliferation through upregulation of cyclin D1 expression, while inhibiting p16 expression, leading to promoting cell proliferation. In the present study, 25–100 μM H_2_O_2_ upregulated the phosphorylated levels of PI3K, Akt, mTOR, and GSK-3β, resulting in activating the PI3K/Akt/mTOR pathway and inhibiting GSK-3β activity; the effect of 50 μM H_2_O_2_ is the strongest. These results further demonstrated that a low dose of H_2_O_2_ possesses growth factor-like characteristics, which could activate the PI3K/Akt pathway. The growth hormones and cytokines are the upstream stimulators of this pathway [17, 25]. SDF-1 could promote MSC survival and migration also by the activation of the PI3K/AKT pathway [9, 10, 18–21]. In addition, low dose H_2_O_2_ directly activated this pathway through diffusion into the cytoplasm as a second messenger may be another activation pathway [17, 25] (Fig. 8). Inversely, we confirmed that a high concentration of H_2_O_2_ induced (300 μM) oxidative stress not only inhibited the PI3K/Akt/mTOR pathway and increased the GSK-3β activity by inhibiting the phosphorylated of these key proteins, but also significantly increased the apoptotic rate of BMSCs. Hence, oxidative stress-induced apoptosis was mediated by the inactivation of the PI3K/Akt/mTOR pathway and increased GSK-3β activity, which was consistent with the literature reports [16, 21, 36]. However, we found that 50 μM H_2_O_2_ preconditioning could reduce the inhibition of the PI3K/Akt pathway induced by oxidative stress through upregulation of the phosphorylation of PI3K, Akt, mTOR, and GSK-3β, and the expression of Bcl-2 in BMSCs, finally, resulting in inhibiting of the mitochondrial apoptosis pathway. Therefore, the protective effect of 50 μM H_2_O_2_ preconditioning might be dependent on the activation of the PI3K/Akt signaling pathway, contributing inhibition of its downstream target GSK-3β activity and upregulation of anti-apoptotic Bcl-2 expression.

The success of stem-cell therapy depends on the migration and survival of the transplanted cells [40, 41]. Therefore, enhancing the migration ability and survival of BMSCs are the key to optimize stem cell therapy in wounds. Based on the vitro experiment, we confirmed that 50 μM H_2_O_2_ maximized the migration and viability of stem cells, thereby hypothesizing that H_2_O_2_ preconditioning may enhance BMSCs homing toward wounds and improve their therapeutic efficacy. As we expected, 50 μM H_2_O_2_ preconditioning for 12 h contributed to more efficient engraftment of transplanted BMSCs into the wounds via enhancement of their migration capacity and increasing their survival by prolonging their retention time in the wounds. This result was consistent with the outcomes of in vitro migration studies in which 50 μM H_2_O_2_-proconditioned BMSCs displayed enhancement of migration and survival.

Numerous studies confirm that transplanted MSCs could promote wound healing [42] and suggest that the differentiation and paracrine signaling have both been implicated as mechanisms by which MSCs improve tissue repair. However, growing evidence indicates that MSC paracrine signaling is the predominant mechanism responsible for enhanced wound repair though accelerating wound closure, enhancing reepithelialization, increasing angiogenesis, and promoting granulation tissue formation [42]. Further studies confirm that the pretreatment of stem cells with cytokines, drugs, and reagents can enhance their paracrine function and therapeutic potential of stem cells thereby improving wound healing [15, 23, 42]. In fact, the therapeutic effect of H_2_O_2_-preconditioned BMSCs was significantly better than that of un-preconditioned BMSCs in terms of an increase in the speedy wound closure rate. The higher VEGF levels and the microvessel density in wound healing tissues indicated the critical role of H_2_O_2_-preconditioned BMSCs in elevating angiogenesis for accelerated wound healing. Taken together, these results indicated that the preconditioning of BMSCs with H_2_O_2_ enhanced their therapeutic potential by increasing their engraftment efficiency in wounds, responsive secretion of VEGF, and facilitating neovascularization, thereby improving the quality of wound healing.

## Conclusions

This study first demonstrated that pretreated BMSCs with 50 μM H_2_O_2_ for 12 h was the optimal preconditioning protocol, which was indicated by maximizing activating the pathways of SDF-1/CXCR4 and PI3K/Akt/mTOR and inhibiting GSK-3β activity, thereby playing a cytoprotective role in oxidative stress-induced BMSC apoptosis. Finally, the data confirmed that BMSCs preconditioned with this protocol could significantly improve the therapeutic potential of transplanted stem cells, which might represent an attractive intervention strategy to promote wound healing.

## Supplementary information

**Additional file 1: Supplement Fig. 1.** The BMSCs have maintained the multipotent differentiation capacity of stem cells even after pretreated with 50 μM H2O2 for 12 h.

## Data Availability

The data that support the findings of this study are available from the corresponding author upon request.

## Abbreviations

CM-DiI: Chloromethyl-1,1-dioctadecyl-3,3,3,3-tetramethylindocarbocyanine perchlorate
BMSCs: Bone marrow mesenchymal stem cells
MSCs: Mesenchymal stem cells
H_2_O_2_: Hydrogen peroxide
VEGF: Vascular endothelial growth factor
SDF-1: Stromal cell-derived factor-1
CXCR4: CXC chemokine receptor 4
PI3K: Phosphoinositide 3-kinase
Akt: Protein kinase B
mTOR: Mammalian target of rapamycin
GSK-3β: Glycogen synthase kinase-3β
ROS: Reactive oxygen species
FCM: Flow cytometric
mPTP: Mitochondrial permeability transition pore

## References

[1] Caplan AI, Correa D (2011). The MSC: an injury drugstore. Cell Stem Cell.

[2] Isakson M, de Blacam C, Whelan D (2015). Mesenchymal stem cells and cutaneous wound healing: current evidence and future potential. Stem Cells Int.

[3] Trounson A, McDonald C (2015). Stem cell therapies in clinical trials: progress and challenges. Cell Stem Cell.

[4] Golchin A, Farahany TZ, Khojasteh A (2019). The clinical trials of mesenchymal stem cell therapy in skin diseases: an update and concise review. Curr Stem Cell Res Ther.

[5] Haider H, Ashraf M (2010). Preconditioning and stem cell survival. J Cardiovasc Transl Res.

[6] Peart JN, Headrick JP (2008). Sustained cardioprotection: exploring unconventional modalities. Vasc Pharmacol.

[7] Cencioni C, Capogrossi MC, Napolitano M (2012). The SDF-1/CXCR4 axis in stem cell preconditioning. Cardiovasc Res.

[8] Askari AT, Unzek S, Popovic ZB (2003). Effect of stromal-cell-derived factor 1 on stem-cell homing and tissue regeneration in ischaemic cardiomyopathy. Lancet.

[9] Marquez-Curtis LA, Janowska-Wieczorek A (2013). Enhancing the migration ability of mesenchymal stromal cells by targeting the SDF-1/CXCR4 axis. Biomed Res Int.

[10] Ceradini D, Anita K, Callaghan M, Tepper O, Bastidas N, Kleinman M, Capla J, Galiano R, Levine J, Gurtner G (2004). Progenitor cell trafficking is regulated by hypoxic gradients through HIF-1 induction of SDF-1. Nat Med.

[11] Xu XZF, Zhang M, Zeng D, Luo D, Liu G, Cui W, Wang S, Guo W, Xing W, Liang H, Li L, Fu X, Jiang J, Huang H (2013). Stromal cell-derived factor-1 enhances wound healing through recruiting bone marrow-derived mesenchymal stem cells to the wound area and promoting neovascularization. Cells Tissues Organs.

[12] Amiri F, Jahanian-Najafabadi A, Roudkenar MH (2015). In vitro augmentation of mesenchymal stem cells viability in stressful microenvironments: in vitro augmentation of mesenchymal stem cells viability. Cell Stress Chaperones.

[13] Haider H, Ashraf M (2008). Strategies to promote donor cell survival: combining preconditioning approach with stem cell transplantation. J Mol Cell Cardiol.

[14] Toma C, Wagner WR, Bowry S (2009). Fate of culture-expanded mesenchymal stem cells in the microvasculature: in vivo observations of cell kinetics. Circ Res.

[15] Chen J, Crawford R, Chen C (2013). The key regulatory roles of the PI3K/Akt signaling pathway in the functionalities of mesenchymal stem cells and applications in tissue regeneration. Tissue Eng Part B Rev.

[16] Le Belle JEON, Paucar AA, Saxe JP, Mottahedeh J, Pyle AD, Wu H, Kornblum HI (2011). Proliferative neural stem cells have high endogenous ROS levels that regulate self-renewal and neurogenesis in a PI3K/Akt-dependant manner. Cell Stem Cell.

[17] Liu J, Finkel T (2013). Stem cells and oxidants: too little of a bad thing. Cell Metab.

[18] De Falco E, Porcelli D, Torella AR (2004). SDF-1 involvement in endothelial phenotype and ischemia-induced recruitment of bone marrow progenitor cells. Blood.

[19] Liu X, Duan B, Cheng Z (2011). SDF-1/CXCR4 axis modulates bone marrow mesenchymal stem cell apoptosis, migration and cytokine secretion. Protein Cell.

[20] Rosova I, Dao M, Capoccia B (2008). Hypoxic preconditioning results in increased motility and improved therapeutic potential of human mesenchymal stem cells. Stem Cells.

[21] Srijaya TC, Ramasamy TS, Kasim NH (2014). Advancing stem cell therapy from bench to bedside: lessons from drug therapies. J Transl Med.

[22] Boopathy AV, Pendergrass KD, Che PL (2013). Oxidative stress-induced Notch1 signaling promotes cardiogenic gene expression in mesenchymal stem cells. Stem Cell Res Ther.

[23] Khatlani T, Algudiri D, Alenzi R (2018). Preconditioning by hydrogen peroxide enhances multiple properties of human decidua basalis mesenchymal stem/multipotent stromal cells. Stem Cells Int.

[24] Pendergrass KD, Boopathy AV, Seshadri G (2013). Acute preconditioning of cardiac progenitor cells with hydrogen peroxide enhances angiogenic pathways following ischemia-reperfusion injury. Stem Cells Dev.

[25] Holmstrom KM, Finkel T (2014). Cellular mechanisms and physiological consequences of redox-dependent signalling. Nat Rev Mol Cell Biol.

[26] Lee BWL, Ghode P, Ong DST (2019). Redox regulation of cell state and fate. Redox Biol.

[27] Rhee SG (2006). Cell signaling. H_2_O_2_, a necessary evil for cell signaling. Science.

[28] Zhang H, Wang P, Zhang X (2019). SDF1/CXCR7 signaling axis participates in angiogenesis in degenerated discs via the PI3K/AKT pathway. DNA Cell Biol.

[29] Maraldi T, Angeloni C, Giannoni E (2015). Reactive oxygen species in stem cells. Oxidative Med Cell Longev.

[30] Youle RJ, Strasser A (2008). The BCL-2 protein family: opposing activities that mediate cell death. Nat Rev Mol Cell Biol.

[31] Denu RA, Hematti P (2016). Effects of oxidative stress on mesenchymal stem cell biology. Oxidative Med Cell Longev.

[32] Chowdhury SKR, Dobrowsky RT, Fernyhough P (2011). Nutrient excess and altered mitochondrial proteome and function contribute to neurodegeneration in diabetes. Mitochondrion.

[33] Melemedjian OK, Asiedu MN, Tillu DV, et al. Targeting adenosine monophosphate-activated protein kinase (AMPK) in preclinical models reveals a potential mechanism for the treatment of neuropathic pain. Mol Pain. 2011;7:70. 10.1186/1744-8069-7-70 PMC3186752 21936900

[34] Nouri F, Nematollahi-Mahani SN, Sharifi AM (2019). Preconditioning of mesenchymal stem cells with non-toxic concentration of hydrogen peroxide against oxidative stress induced cell death: the role of hypoxia-inducible factor-1. Adv Pharm Bull.

[35] Wang K, Zhang T, Dong Q (2013). Redox homeostasis: the linchpin in stem cell self-renewal and differentiation. Cell Death Dis.

[36] Wullschleger S, Loewith R, Hall MN (2006). TOR signaling in growth and metabolism. Cell.

[37] Huang H, Cui W, Qiu W (2015). Impaired wound healing results from the dysfunction of the Akt/mTOR pathway in diabetic rats. J Dermatol Sci.

[38] Jang MW, Yun SP, Park JH (2012). Cooperation of Epac1/Rap1/Akt and PKA in prostaglandin E (2) -induced proliferation of human umbilical cord blood derived mesenchymal stem cells: involvement of c-Myc and VEGF expression. J Cell Physiol.

[39] Juhaszova M, Zorov DB, Yaniv Y (2009). Role of glycogen synthase kinase-3β in cardioprotection. Circ Res.

[40] Ganju RK, Brubaker SA, Meyer J (1998). The alpha-chemokine, stromal cell-derived factor-1alpha, binds to the transmembrane G-protein-coupled CXCR-4 receptor and activates multiple signal transduction pathways. J Biol Chem.

[41] Zaruba MM, Franz WM (2010). Role of the SDF-1-CXCR4 axis in stem cell-based therapies for ischemic cardiomyopathy. Expert Opin Biol Ther.

[42] Lee DE, Ayoub N, Agrawal DK (2016). Mesenchymal stem cells and cutaneous wound healing: novel methods to increase cell delivery and therapeutic efficacy. Stem Cell Res Ther.

