# Supplementary material for: Optimal H2O2 preconditioning to improve bone marrow mesenchymal stem cells’ engraftment in wound healing
Source: Stem Cell Res Ther. 2020 Oct 8;11:434. doi: 10.1186/s13287-020-01910-5 (PMC7545926; doi:10.1186/s13287-020-01910-5)
Supplement: Supplementary file 1 — Additional file 1: Supplement Fig. 1. The BMSCs have maintained the multipotent differentiation capacity of stem cells even after pretreated with 50 μM H2O2 for 12 h. [file 13287_2020_1910_MOESM1_ESM.docx]

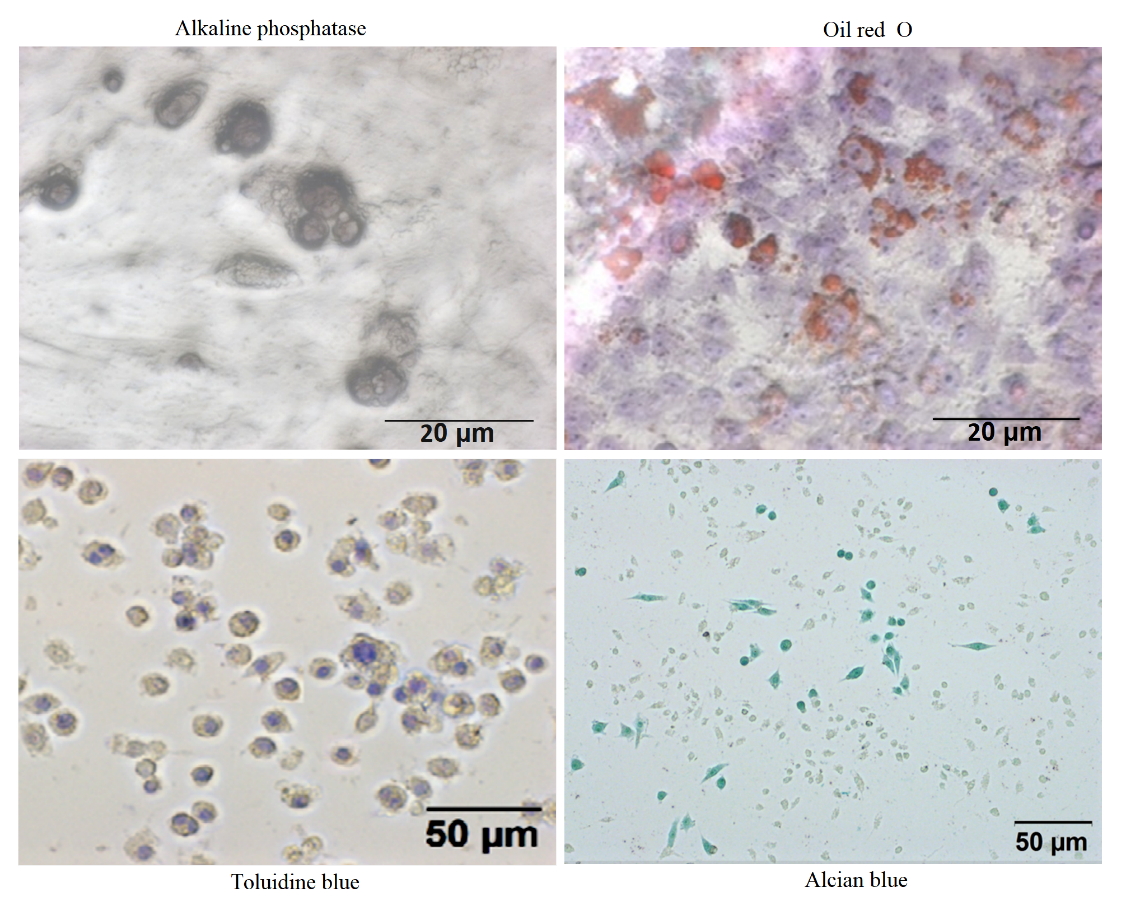


Supplement Fig.1

The BMSCs have maintained the multipotent differentiation capacity of stem cells even after pretreated with 50 μM H_2_O_2_ for 12 h. Morphology was visualized with phosphatase (Osteogenic differentiation), Oil red O (Adipogenic differentiation), Toluidine blue and Alcian blue (chondrocytic differentiation) staining. Osteoblasts, chondrocytes and adipocytes derived from H_2_O_2_-treated BMSCs experienced morphological changes.
